# Using an Inbred Horse Breed in a High Density Genome-Wide Scan for Genetic Risk Factors of Insect Bite Hypersensitivity (IBH)

**DOI:** 10.1371/journal.pone.0152966

**Published:** 2016-04-12

**Authors:** Brandon D. Velie, Merina Shrestha, Liesbeth Franҫois, Anouk Schurink, Yohannes G. Tesfayonas, Anneleen Stinckens, Sarah Blott, Bart J. Ducro, Sofia Mikko, Ruth Thomas, June E. Swinburne, Marie Sundqvist, Susanne Eriksson, Nadine Buys, Gabriella Lindgren

**Affiliations:** 1 Department of Animal Breeding & Genetics, Swedish University of Agricultural Sciences, Uppsala, Sweden; 2 Research Group Livestock Genetics, Department of Biosystems, KU Leuven, Leuven, Belgium; 3 Animal Breeding and Genomics Centre, Wageningen University, Wageningen, the Netherlands; 4 School of Veterinary Medicine & Science, University of Nottingham, Leicestershire, United Kingdom; 5 Exmoor Pony Society, Cullompton, United Kingdom; 6 Animal DNA Diagnostics Ltd, Cambridgeshire, United Kingdom; 7 Animal Health Trust, Newmarket, United Kingdom; 8 Östra Greda Research Group, Borgholm, Sweden; CSIRO, AUSTRALIA

## Abstract

While susceptibility to hypersensitive reactions is a common problem amongst humans and animals alike, the population structure of certain animal species and breeds provides a more advantageous route to better understanding the biology underpinning these conditions. The current study uses Exmoor ponies, a highly inbred breed of horse known to frequently suffer from insect bite hypersensitivity, to identify genomic regions associated with a type I and type IV hypersensitive reaction. A total of 110 cases and 170 controls were genotyped on the 670K Axiom Equine Genotyping Array. Quality control resulted in 452,457 SNPs and 268 individuals being tested for association. Genome-wide association analyses were performed using the GenABEL package in R and resulted in the identification of two regions of interest on Chromosome 8. The first region contained the most significant SNP identified, which was located in an intron of the DCC netrin 1 receptor gene. The second region identified contained multiple top SNPs and encompassed the *PIGN*, *KIAA1468*, *TNFRSF11A*, *ZCCHC2*, and *PHLPP1* genes. Although additional studies will be needed to validate the importance of these regions in horses and the relevance of these regions in other species, the knowledge gained from the current study has the potential to be a step forward in unraveling the complex nature of hypersensitive reactions.

## Introduction

Often referred to as allergic eczema or allergic dermatitis, inflammation of the skin resulting from a hypersensitive reaction that is atypical within a population occurs not only in humans, but in a wide range of species from domestic dogs to hippopotamuses [1–3]. Although widely accepted as a condition where susceptibility is determined by both genetic and environmental factors, there is still a considerable amount to learn regarding the complex roles genes play in these hypersensitive reactions [2,4–6]. Major histocompatibility complex (MHC) genes as well as genes affecting immune response, epithelial barriers, and tissue remodeling have all been suggested as important. However, despite an increased awareness of the aetiology of these reactions, the prevalence of allergic dermatitis and other hypersensitive reactions continues to rise in many species [6–10]. Consequently, the need for additional research exploring the roles genes play in these types of reactions is warranted as new research is likely to contribute to a better understanding of both the genetic and environmental risk factors associated with these conditions.

To date, most research investigating the genetic contribution to the manifestation of allergic dermatitis has taken place in humans [3,5,8,9]. However, given the population structures and higher levels of linkage disequilibrium (LD) in many domestic animal species, animal models likely provide a more advantageous avenue for genetic research into hypersensitivity. Selection for specific characteristics in most domestic animal species has resulted in low within breed genetic variation, thus lessening the amount of genetic markers needed to achieve powerful genome scans and reducing the number of variants as candidate mutations. Although less commonly used as an animal model, horses in particular provide a unique opportunity to further increase our understanding of the genetic aetiology of hypersensitive reactions across species.

Insect bite hypersensitivity (IBH), an allergic recurrent seasonal dermatitis, is the most common allergic disease in horses with the worldwide prevalence in some breeds as high as 60% [11–13]. Frequently referred to as summer eczema, IBH involves IgE-mediated, type I hypersensitivity with release of histamine and other inflammatory mediators such as basophils and mast cells. Cell-mediated, type IV hypersensitivity has also been suggested as a potential contributor to the pathogenesis of the disease [11,14]. Resulting from the bites of insects predominantly from the genus *Culicoides* and characterized by pruritic dermatosis, IBH severely reduces the welfare of affected horses [12,15,16]. Although a polygenetic mode of inheritance has been shown for IBH, genomic research on the disease has thus far been limited to candidate gene approaches and low density genotyping arrays [12,13,15,17–19]. While these studies have undoubtedly provided valuable information regarding the genes likely to be involved in the expression of IBH, there is still much about this hypersensitive reaction and its underlying biology that is not fully understood. That being said, the recent development of the high density Axiom Equine Genotyping Array provides the opportunity for a more comprehensive analysis of the genetic contribution to IBH.

Exmoor ponies, an old breed of horse native to the British Isles, are known to express IBH, with the severity of the disease and number affected representing a significant problem within the breed [7,20]. Considered threatened or endangered by many organizations, Exmoor ponies are ideally suited for genome-wide association analyses exploring hypersensitive reactions such as IBH [21–23]. Their small population likely corresponds with very low within breed genetic variation allowing for a straightforward case-control design [24]. As a result, both the effects and likelihood of multiple subgroups within the population differing in both allele frequency and disease prevalence is minimal [12]. Thus, a more accurate assessment of the relationship between genomic regions and IBH is achievable.

The aim of the current study was to capitalize on these advantages and identify genomic regions associated with IBH in Exmoor ponies using the largest genome-wide association analysis for IBH in horses to date [12,13,19]. When one considers the prevalence of IBH in horse breeds as well as the increasing occurrence of many forms of allergic dermatitis in humans and other species, it is possible that common genetic components across breeds and potentially even species may be identified [3,9]. An increased knowledge of the genes involved in the manifestation of IBH in horses is expected to not only improve prevention, diagnosis, and treatment of IBH in horses, but may also broaden our understanding of the biology underlying type I and type IV hypersensitive reactions across species.

## Materials and Methods

### Sample collection & phenotyping

**Fig 1:**
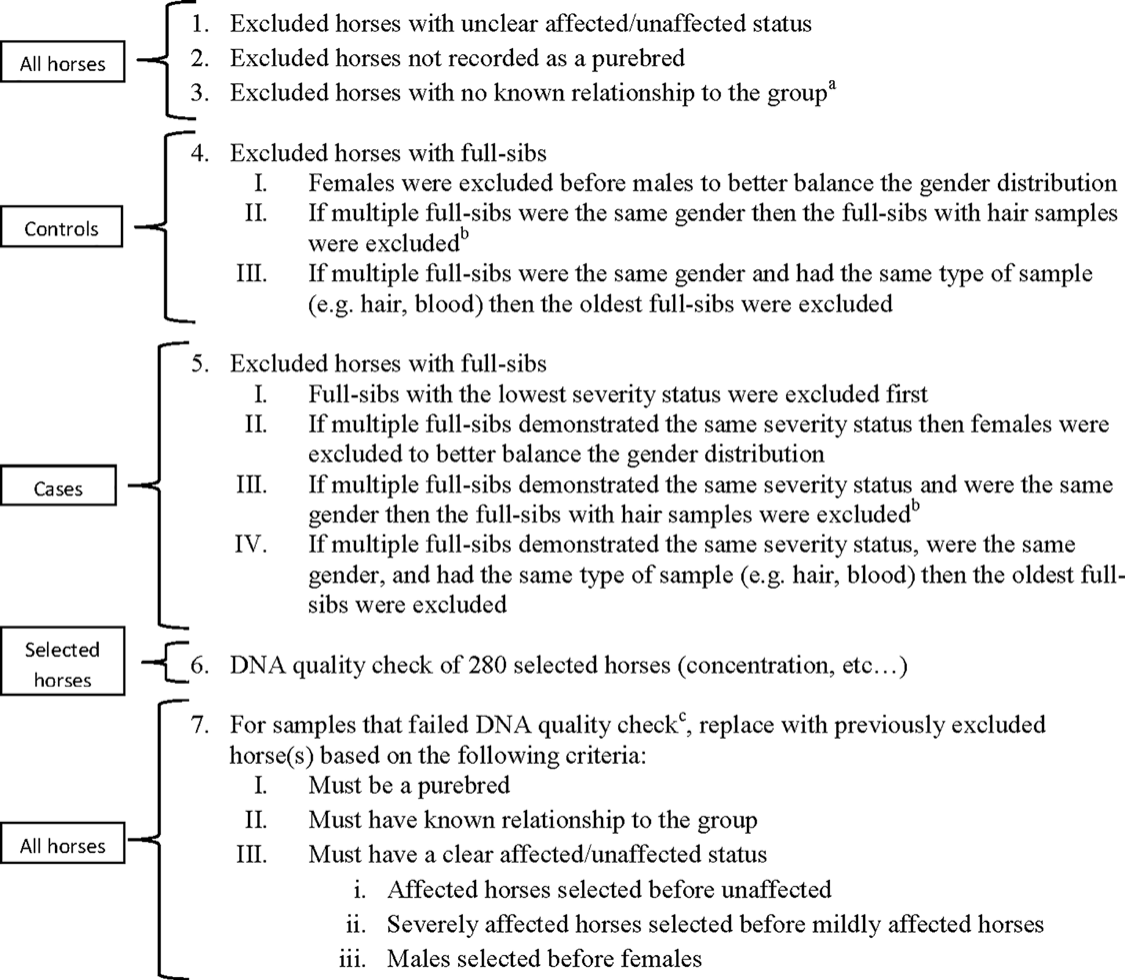
Exmoor Pony Genotyping Exclusion and Inclusion Procedure. ^a^Any horse that did not have complete pedigree information for 4 generations was considered to have no known relationship to the group. ^b^For DNA quality reasons, blood samples were preferred over hair samples and younger horses were selected over older horses. ^c^Samples from two horses yielded insufficient concentrations of DNA for genotyping and were subsequently replaced

Samples from 336 Exmoor ponies were collected between 2008 and 2011 through an open call to owners via the Exmoor pony society and online postings. An owner questionnaire designed to determine IBH status and severity, as well as detail the environmental conditions of each horse, was used to phenotype each individual. Owners of the Exmoor ponies gave permission for their animals to be used in the study and the study was approved by the Ethics Committee for Animal Experiments in Uppsala, Sweden [Number: C 121/14]. A description of how IBH severity scores were assigned is shown in Table 1. Pedigree information (4 generations) for each horse was then obtained from the Exmoor Pony Society database. Based on this information the average relatedness of each individual to the group was estimated using the genetic software Contribution, Inbreeding, Coancestry (CFC) [25]. Any horse with missing pedigree information and/or an unclear IBH phenotype was removed from the study (n = 26). Horses were categorized as either IBH affected (cases) or IBH unaffected (controls) and assigned an IBH severity score (Table 1). 280 horses were then selected for genotyping according to the protocol detailed in Fig 1. Although older horses were more likely to have had longer exposure times, samples from younger horses were prioritized. This prioritization directly resulted from many of the older horses having died (natural causes) prior to the start of the study and the potential need for additional samples of horses used in the study later in the analyses.

**Table 1:** Insect bite hypersensitivity severity scale.

IBH severity score assigned	Questionnaire options
0	Healthy: unaffected; no signs of IBH
1	Mild: not affected if preventative measures are taken
2	Moderate: shows itching even when preventative measures are taken
3	Severe: shows wounds even when preventative measures are taken

### DNA isolation

Deoxyribonucleic acid was prepared from the hair roots using a standard hair-preparation procedure. Briefly, 186 μL Chelex 100 Resin (Bio-Rad Laboratories, Hercules, CA) and 14 μL of proteinase K (20 mg/mL; Merck KgaA, Darmstadt, Germany) were added to the sample. The mix was incubated at 56°C for 2 h and the proteinase K was inactivated for 10 min at 95°C. For DNA preparation from blood samples, 200 μL of blood was used and isolated on the Qiasymphony instrument using the Qiasymphony DSP DNA mini kit (Qiagen, Hilden, Germany). Samples for two horses failed to meet the DNA quality requirements for genotyping and were replaced (Fig 1). Descriptive statistics of the final horses selected for genotyping are shown in Table 2. The final horses selected represented 107 sires and 226 dams.

**Table 2:** Descriptive statistics for the genotyped horses.

	Controls	Cases			Total
IBH severity^a^	0	1	2	3	
Males	52	7	18	17	**94**
Females	118	18	22	28	**186**
**Total**	**170**	**25**	**40**	**45**	**280**

^a^IBH severity scores: 0 = healthy, unaffected; 1 = mildly affected, not affected if preventative measures are taken; 2 = moderately affected, shows itching even when preventative measures are taken; 3 = severely affected, shows wounds even when preventative measures are taken

### Genotyping and quality control

Prior to quality control (QC) the SNP data set consisted of 280 individuals genotyped using the 670K Axiom Equine Genotyping Array. Average relatedness of the individuals genotyped was 0.23. Iterative QC was performed with the GenABEL package in R to remove poorly genotyped and noisy data using the following thresholds: minor allele frequency (MAF) (<0.5%), missing genotypes per single nucleotide polymorphism (SNP) (>10%), missing SNPs per sample (>10%), and Hardy-Weinberg equilibrium (HWE) (first QC p<1e^-10^; second QC FDR<0.2 in IBH controls only) [26].

### Genome-wide association analysis

**Fig 2:**
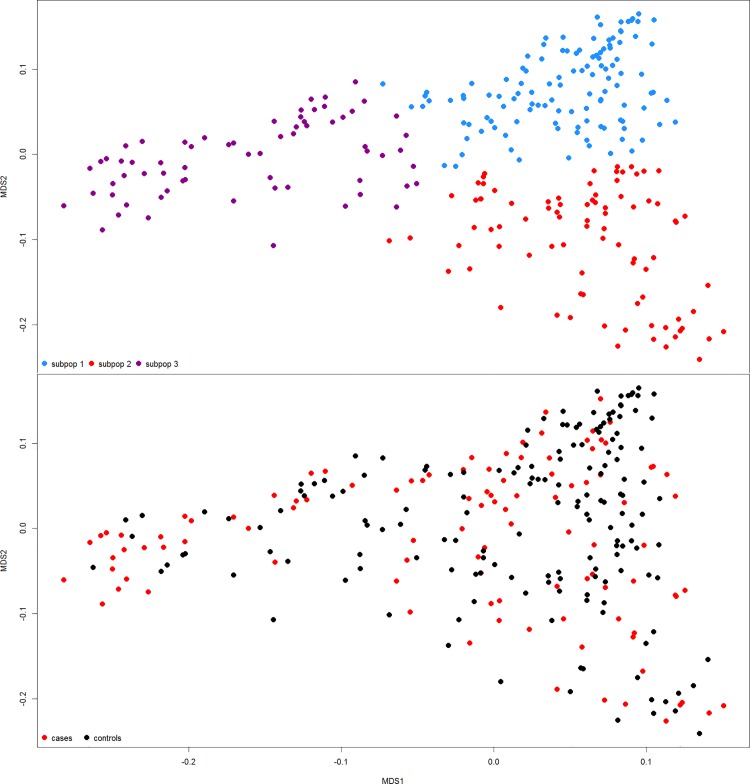
Visualization of population stratification and distribution of cases and controls across the 3 subpopulations

Genome-wide association (GWA) analyses were performed using the GenABEL package in R (R Development Core Team 2011). An autosomal genomic kinship matrix was computed and standard K-means clustering was performed. To determine the number of clusters (subpopulations), K-means clustering with K = {1,2,…,10} were completed. For each iteration the sum of within-cluster sums of squares (∑WCSS) was calculated and plotted vs. K. The number of clusters corresponding with the first inflection point (K = 3) was then chosen to define the subpopulations [27]. No outliers were apparent on the multidimensional scaling (MDS) plot. A visualization of the genomic-kinship matrix and subpopulations using MDS are shown in Fig 2. To avoid spurious associations that may arise with unusual allele frequency differences between sub-populations, multiple methods to correct for population stratification were applied [28].

GWA analyses of IBH status classified as cases and controls were performed using both a structured association approach (“qtscore” function in GenABEL) and a principal component approach (“egscore” function). GWA analyses of IBH severity scores were performed using both a mixed model-structured association approach (“mmscore” function) and an additional principal component approach. Preliminary analyses did not indicate a significant effect of gender on IBH thus it was not included as a co-variant in the final analyses. Information pertaining to the environment of the horse was also excluded from all analyses as questionnaire responses regarding environment descriptions were inconsistent across respondents. After 100,000 permutations, associations with individual SNPs were considered genome-wide significant for p-values below 0.05. Due to computational limitations, only 5,000 permutations were performed for the mixed model-structured association analysis. However, the genome-wide significance threshold remained at *P*<0.05. Regions of interest were defined as any 1MB region that contained multiple SNPs below the suggested genome-wide significance threshold (*P* < 1x10^-5^) for two or more of the GWA approaches performed on the dataset.

## Results

**Fig 3:**
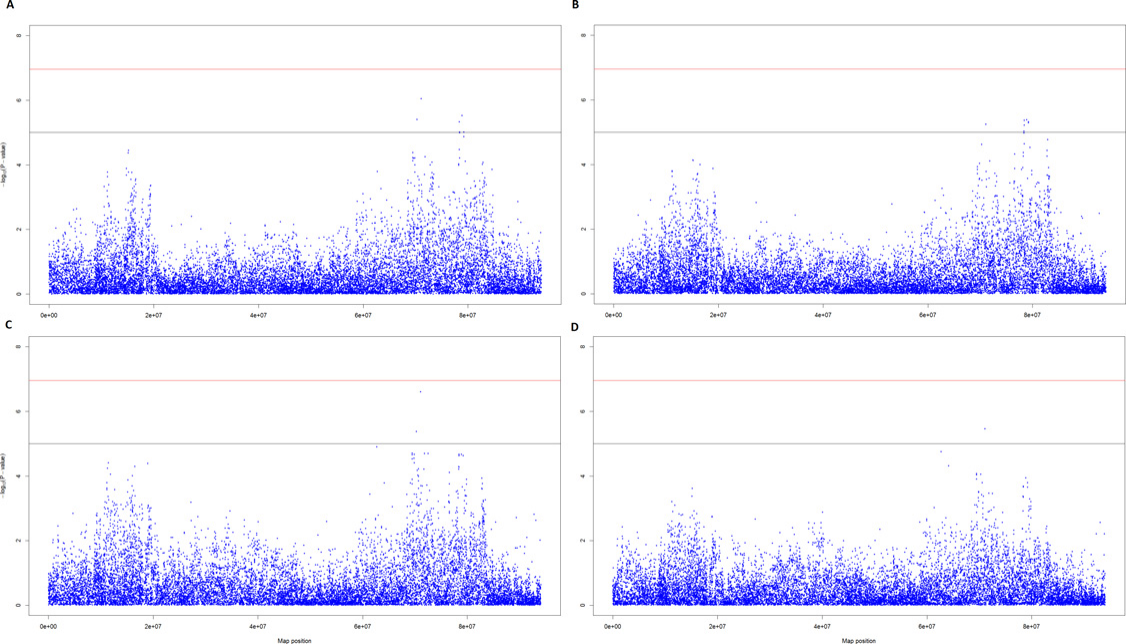
Manhattan plots for Chromosome 8 (ECA8). ^a^Manhattan plot based on the results of the cases and controls structured association analysis. ^b^Manhattan plot based on the results of the cases and controls principal component analysis. ^c^Manhattan plot based on the results of the principal component analysis of IBH severity. ^d^Manhattan plot based on the results of the mixed model-structured association analysis of IBH severity. ^e^The red line indicates the Bonferroni-corrected significance threshold; the black line indicates the threshold for suggestive SNPs (*P* < 1x10^-5^).

**Fig 4:**
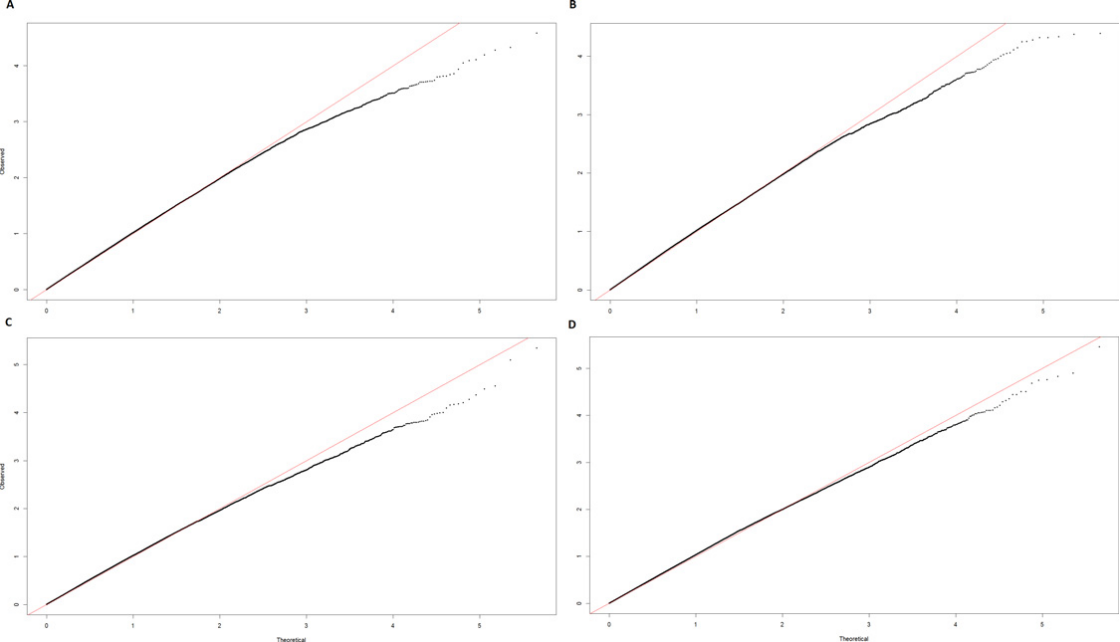
QQ plots for Chromosome 8 (ECA8). ^a^Corrected QQ plot for the cases and controls structured association analysis; uncorrected λ = 1.3659. ^b^Corrected QQ plot for the cases and controls principal component analysis; uncorrected λ = 1.2599. ^c^Corrected QQ plot for the principal component analysis of IBH severity; uncorrected λ = 1.2657. ^d^Corrected QQ plot for the mixed model-structured association analysis of IBH severity; uncorrected λ = 1.0222.

**Fig 5:**
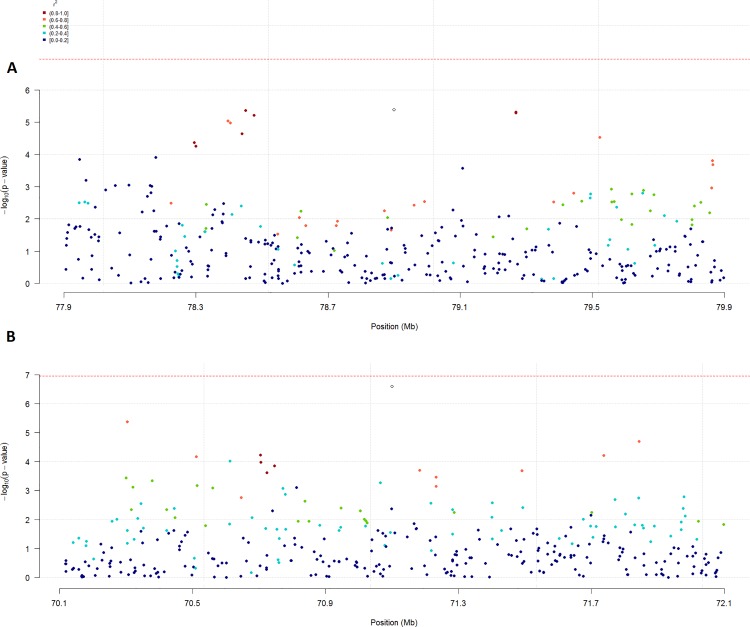
Linkage disequilibrium patterns relative to markers AX-104130346 and AX-104330407. ^a^Linkage disequilibrium pattern relative to marker AX-104330407 on a Manhattan plot based on the results of the cases and controls principal component analysis. ^b^Linkage disequilibrium pattern relative to marker AX-104130346 on a Manhattan plot based on the results of the principal component analysis of IBH severity

Following QC, 452,457 SNPs and 268 individuals were tested for association. No single SNP demonstrated genome-wide significance in any of the four GWA analyses performed. However, 2 regions of interest on Chromosome 8 (ECA8) were apparent with a single SNP, AX-104130346, located at 71,065,803 bp resulting in the lowest p-value (*P*_unadjusted_) in 3 out of the 4 analyses and bordering on genome-wide significance (*P*_genome-wide_) in two (Table 3). This SNP has alleles T and C, with a MAF (C) of 0.34 and does not deviate from HWE (exact HWE test P-value = 0.174). The genotype frequencies of AX-104130346 in cases and controls are shown in Table 4. The first region of interest (chr.8: 70,269,986–71,065,803) included a SNP (AX-104130346) located in the DCC netrin 1 receptor gene (*DCC*) (Cunningham et al. 2015). The second region of interest (chr.8: 78,377,554–78,880,555) consisted of 4 SNPs and contained 5 genes: *PIGN*, *KIAA1468*, *TNFRSF11A*, *ZCCHC2*, and *PHLPP1* (Table 3; Table 5). Manhattan Plots and QQ plots resulting from each of the analyses for these regions are shown in Fig 3, Fig 4 and Fig 5.

**Table 3:** Unadjusted and genome-wide *p*-values for the four GWA analyses performed.

SNP	ECA	Position	*P*_unadjusted_				*P*_genome-wide_			
			SA (case/control)	PC (case/control)	PC (severity)	MMSA (severity)	SA (case/control)	PC (case/control)	PC (severity)	MMSA (severity)
AX-103266754	1	100460873	8.546e-6				0.512			
AX-104707726	1	100649241	6.652e-6				0.438			
AX-103372605	2	116550518				1.438e-5				0.956
AX-103869604	4	546104	4.119e-6				0.314			
AX-103676516	4	16228799			2.908e-6				0.414	
AX-102955701	6	55403540				8.494e-6				0.887
AX-103802842	7	36418118				8.370e-6				0.883
AX-104843731	8	62711228				5.348e-6				0.788
**AX-104531882**	**8**	**70269986**	**3.924e-6**		**4.205e-6**		**0.303**		**0.515**	
**AX-104130346**	**8**	**71065803**	**8.982e-7**	**5.674e-6**	**2.499e-7**	**2.313e-6**	**0.086**	**0.431**	**0.060**	**0.560**
**AX-103982426**	**8**	**78377554**	**4.722e-6**	**9.260e-6**			**0.346**	**0.581**		
AX-103716604	8	78430916		4.289e-6				0.354		
AX-103206537	8	78456162		6.088e-6				0.451		
**AX-104330407**	**8**	**78880555**	**3.001e-6**	**4.139e-6**			**0.246**	**0.345**		
AX-102952650	8	79249465		5.090e-6				0.401		
AX-104594806	8	79249580		4.836e-6				0.387		
AX-104273278	9	9674307				1.061e-5				0.920
AX-103496042	9	26970670				1.393e-5				0.954
AX-103054421	16	58688991			7.360e-6				0.679	
AX-103679698	16	59338023			7.745e-6				0.693	
AX-104503315	16	59962819			7.156e-6				0.670	
AX-104922413	16	62458479	1.998e-6				0.176			
AX-104674779	16	63068497			5.509e-6				0.593	
AX-104295622	16	65267154	2.285e-6				0.197			
AX-104129425	16	79101000				8.224e-6				0.880
AX-103533051	20	29990169				1.345e-5				0.950
AX-103683812	20	33325386		6.223e-6				0.457		
AX-104146721	20	33542125		8.377e-6				0.549		
AX-103554518	20	49561945	8.964e-6				0.527			
AX-104347157	24	30622320			2.442e-6				0.369	
AX-104881761	24	30624192			5.083e-7				0.111	
AX-102975270	24	30626293			6.686e-6				0.651	
AX-104585909	26	14984244				2.006e-5				0.982
AX-103795343	34	1727788		5.090e-6				0.401		

SA = structured association; PC = principal component; MM = mixed model

SNPs present in the top 10 in multiple GWA analyses are listed in bold

**Table 4:** Genotype frequencies, stratified by IBH affected status, for SNP AX-104130346 following quality control.

	Genotype frequency			N
	T/T	T/C	C/C	
Controls	0.59	0.31	0.10	163
Cases	0.25	0.56	0.19	105

**Table 5:** Descriptive statistics for the top SNPs located within each region of interest.

SNP	Chr	Position	Minor allele	Minor allele frequency	Effect of minor allele^a^
AX-104531882	8	70269986	T	0.44	0.630–2.215
AX-104130346	8	71065803	C	0.34	0.035–2.281
AX-103982426	8	78377554	C	0.25	0.045–2.415
AX-103716604	8	78430916	A	0.21	0.0534
AX-103206537	8	78456162	C	0.21	0.0525
AX-104330407	8	78880555	A	0.21	0.053–2.572

^a^The range of the minor allele effect (difference from the mean) is provided for top SNPs in multiple analyses

## Discussion

Much like autoimmune diseases, incidences of type I hypersensitive reactions have drastically increased over the past few decades [6]. Despite multiple attempts to unravel the genetic contributions both within and across species, the genetic etiology of many of these allergy related diseases remains unresolved. Using horses as a model organism, the current study explored the genetic background of a hypersensitive reaction to insect bites in Exmoor ponies and ultimately identified two regions of potential importance on Chromosome 8 (ECA8).

Of particular interest is the region containing the SNP that resulted in the lowest p-value in 3 out of the 4 analyses performed (Table 3). The SNP, AX-104130346, occurs in an intron of the *DCC* gene, a gene whose corresponding protein has been associated with apoptosis and functions as a tumor suppressor [29]. In humans, the resulting protein has also been observed as mutated or down-regulated in certain types of cancers [29]. This is particularly of note when one considers that hypersensitivity is, by definition, a harmful immune response against usually harmless antigens [6]. Cancers are invasive growths; therefore, it is logical that the body’s immune response would need to be suppressed in order for a cancer to metastasize, hence the down-regulation of a tumor suppressing protein [30]. It is possible that the reverse occurs in horses with IBH, ultimately culminating in a hypersensitive reaction resulting from the up-regulation of a protein associated with the body’s overactive immune response.

In addition to the genomic region described above we found a region of interest with 4 SNPs on ECA8 that encompassed a genomic region approximately 503kb in length. Within this region was SNP AX-104330407, a SNP that resulted in the lowest p-value in one of the GWA analyses and was in the top 10 results of another. While the region consists of five genes, *TNFRSF11A* stands out as a potential candidate gene. Not only is *TNFRSF11A* the closest gene to SNP AX-104330407, but screening for *TNFRSF11A* has already been suggested as a potential diagnostic test for autoinflammatory disorders in humans [31]. Given that autoinflammatory diseases and hypersensitive reactions are both caused by dysfunction of the immune system, genetic similarities between the two are highly probable and make further exploration of this region and gene warranted [6].

Although previous studies exploring IBH have shown fluctuating levels of significance for regions on Chromosomes 3, 9, 11, 20, and 27, no genes on ECA8 have previously been identified as important for susceptibility to IBH [12,13,19]. Despite the fact that both MHC genes and non-MHC genes are likely to be involved in the manifestation of IBH, the current study was unable to decisively support previously identified MHC class II regions as significantly associated with IBH [7,18]. While the exact reasons for this are presently unknown, it has been suggested that MHC genes may not necessarily affect the overall risk of developing a hypersensitive reaction, but may in fact influence what an individual becomes allergic to. This is demonstrated by the genetic differences between a rye grass allergy (associated with HLA-DR3) and a birch pollen allergy (associated with HLA-DR5) in humans [6]. However, four SNPs on Chromosome 20 did appear in the list of SNPs with the lowest p-values, likely warranting further exploration of Chromosome 20. No other previously reported regions were among the list of SNPs with the lowest p-values.

Lack of correspondence with previous GWA studies for the regions on ECA8 may also have resulted from use of the 50K and 70K genotyping arrays in earlier studies [12,13,19]. Past studies would likely have been predominantly focused on relatively common variants across breeds, while the current study was able to explore significantly more variants across the genome. A substantial advantage when one considers that most of the heritability for IBH has been unexplained by the low density array GWAS [12,13,15,19]. Although no single SNP demonstrated genome-wide significance (GWS) in the current study, it is important to note that insect bite hypersensitivity is widely accepted as a highly complex, multi-factorial disease that can be difficult to diagnose when manifestation is not severe. Though not considered to be a significant factor in the current study, the power of studies exploring complex diseases can potentially be weakened when individuals reported as controls exhibit a mild, often unnoticed form of the disease. This in-turn negatively affects the likelihood of a SNP significantly associated with the disease demonstrating GWS. However, it is critical to keep in mind that associations with borderline GWS have been shown to be successfully replicated 73% of the time with many of them achieving substantially lower p-values when additional data are obtained [32].

As such, it is important to put the results of the current study in context. Taking the position that if a genomic region or SNP was truly important in the expression of IBH, it would remain as one of the top regions or SNPs regardless of the methodology chosen, the current study reports the results of 4 different approaches [28]. While a SNP may not reach GWS or even remain as the top potential marker in all approaches, any potentially important SNPs or regions would feasibly appear in all analyses whereas spurious associations would be less likely to endure across each analysis. Although this does not necessary exclude other SNPs or regions as potentially significant in the expression of IBH, it goes a long way in strengthening the confidence in any potential candidate genes and genomic regions identified in the study.

While the results of the current study will still require validation in a large independent data set, they potentially provide further insight into the genetic etiology of IBH in horses. By capitalizing on the newly available high density equine genotyping array and a highly inbreed breed of horse with a well-documented susceptibility to hypersensitive reactions, the current study identifies at least one candidate gene that has previously not even been suggested as important in the manifestation of IBH. The supplementary knowledge gained from this study together with other IBH studies in horses will conceivably bring researchers closer to fully understanding the biology underlying type I and type IV hypersensitive reactions in not only horses, but other species as well.
